# A 64-year old man presenting with carotid artery occlusion and corticobasal syndrome: a case report

**DOI:** 10.1186/1752-1947-5-357

**Published:** 2011-08-09

**Authors:** Marc Engelen, Dunja Westhoff, Jan de Gans, Paul J Nederkoorn

**Affiliations:** 1 Department of Neurology, H2.216, Academic Medical Center, University of Amsterdam, PO Box 22660, 1100 DD Amsterdam, The Netherlands

## Abstract

**Introduction:**

Magnetic resonance imaging of the brain in patients with corticobasal degeneration typically shows focal or asymmetric atrophy, usually maximal in the frontoparietal cortex. Many patients who are diagnosed with corticobasal degeneration using current diagnostic criteria do not have classical corticobasal degeneration pathology. Our case is remarkable for the fact that the symptoms and the characteristic magnetic resonance imaging appearance were typical for corticobasal degeneration. However, we were quite convinced that the clinical picture had a vascular etiology. Only a few cases have been reported where the presumed cause for the corticobasal syndrome was multiple brain infarctions bilaterally.

**Case presentation:**

A 64-year-old Caucasian man visited a neurologist because of profound asymmetric sensory and motor disturbances. A magnetic resonance imaging scan of his brain revealed occlusion of his internal carotid artery on the left side with multiple vascular lesions in his left hemisphere and notable atrophy of mainly the left parietal and frontal cortex.

**Conclusion:**

We describe a patient with corticobasal syndrome caused by multiple infarctions, probably caused by emboli of the carotid stenosis. This patient illustrates the fact that the word 'syndrome' should be preferred above 'degeneration' in the name of this disease.

## Introduction

Corticobasal degeneration (CBD) was formerly considered to be a well-defined clinicopathological entity. The classic description of CBD includes clumsiness and loss of function of one hand due to a combination of frontoparietal and basal ganglia sensorimotor dysfunction [1]. However, many patients who are diagnosed using current diagnostic criteria do not have classical corticobasal degeneration pathology [2]. Therefore it is now customary to diagnose corticobasal syndrome (CBS) during life, and refer to the classical pathology as CBD. CBS can be caused by classical CBD pathology, but also by the pathology of progressive supranuclear palsy, frontotemporal lobe degeneration or even Alzheimer's [3]. A few cases have been reported where the presumed cause of CBS was multiple brain infarctions bilaterally [4]. Magnetic resonance imaging (MRI) of the brain in patients with CBS typically shows focal or asymmetric atrophy, usually maximal in the frontoparietal cortex.

## Case presentation

A 64-year-old Caucasian man experienced sudden cramping of the toes of his right foot, and simultaneous weakness and numbness of his right leg. This lasted for approximately 20 minutes, after which he completely recovered. These incidents recurred, increasing in frequency for several weeks. At first, he fully recovered after each episode. Some weeks later, he noticed a persisting numbness of both his right leg and hand. Walking became more difficult because of roaming and clumsiness of his right leg. About 10 months later, he visited a general practitioner and was referred to a neurologist. At that time he experienced gait difficulty and numbness of his right arm and leg. His previous medical history was remarkable for hypertension and he is a cigarette smoker. He uses metoprolol but no other medication. He had no significant family history of neurological disease.

**Figure 1:**
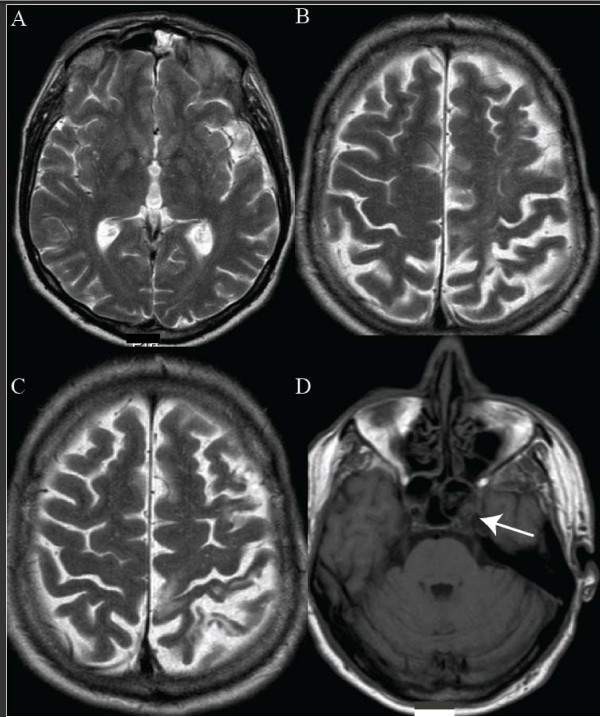
**Atrophy of the left frontoparietal lobe, with extensive gliosis (A, B, C; T2-weighted MRI)**. The left internal carotid artery is occluded, since there is no flow void (D; T1-weighted MRI).

On examination there was flattening of the nasolabial fold on the right side of his face. The fine motor skills of his right arm were impaired. There was clearly impaired two-point discrimination on both his right arm and leg, while position-, movement-, and vibration-sense were intact. There was hyperpathia of his right leg and arm. Ataxia of his right leg was noted, not improving with visual correction. Deep tendon reflexes were higher in his right leg, with a Babinski sign. There was a hen's gait on his right side. An MRI scan of his brain performed in the referring hospital revealed occlusion of his internal carotid artery on the left side with multiple vascular lesions in the left hemisphere and notable atrophy of mainly the left parietal and frontal cortex (Figure 1). We started prophylactic treatment with aspirin, dipyridamole and simvastatin. We urged our patient to stop smoking.

## Discussion

Our patient fulfilled the criteria for CBS with a profound asymmetric presentation, with dystonia of the right foot, cortical sensory disturbance (with profoundly impaired two-point discrimination) and pyramidal tract syndrome. MRI of the brain showed left frontoparietal atrophy with multiple subcortical hyperintensities. Revision of the MRI scan by our neuroradiologist revealed occlusion of the left internal carotid artery.

Usually, the onset of CBS is insidious. This patient described an acute onset of symptoms, suggesting a vascular origin. After that there were a few instances of fluctuating deficits, but eventually there was residual impairment as described. There does not appear to have been any further progression over the last few months. The clinical picture and the evolution of symptoms seem compatible with a presumed vascular cause in this patient.

A few cases of a presumed vascular origin of CBS have been reported [4,5] (Table 1), however our case is remarkable for the fact that it is associated with marked frontoparietal atrophy on brain imaging, therefore also mimicking the characteristic MRI appearance. It is likely that multiple strokes resulted in atrophy and gliosis.

**Table 1:** Previously published cases

Article	Patient	Diagnosis	Symptoms
Kim *et al. *[4]	75-year-old woman	extensive cortical vascular-ischemic lesions	Progressive symptoms of: - dementia - asymmetric parkinsonism - apraxia - action myoclonus - focal hand dystonia

Kreisler *et al. *[5]	5 women, aged between 64 and 77 years	extensive vascular lesions	Progressive symptoms of: - asymmetric parkinsonism - apraxia - focal action myoclonus - focal dystonia - cortical sensory loss - alien limb phenomenon

## Conclusion

We describe a patient with CBS caused by multiple infarctions, probably caused by emboli of his carotid stenosis. This patient illustrates the fact that the word 'syndrome' should be preferred above 'degeneration' in the name of this disease.

## Consent

Written informed consent was obtained from the patient for publication of this case report and any accompanying images. A copy of the written consent is available for review by the Editor-in-Chief of this journal.

## Competing interests

The authors declare that they have no competing interests.

## Authors' contributions

DW and ME wrote this case report. PJN and JdG revised it critically. All authors read and approved the final manuscript.
